# Maternal Territoriality Achieved Through Shaking and Lunging: An Investigation of Patterns in Associated Behaviors and Substrate Vibrations in a Colonial Embiopteran, *Antipaluria urichi*

**DOI:** 10.1673/031.013.8201

**Published:** 2013-09-02

**Authors:** Khaaliq A. Dejan, John M. Fresquez, Annika M. Meyer, Janice S. Edgerly

**Affiliations:** Department of Biology, Santa Clara University, Santa Clara, CA 95053, USA

**Keywords:** colonial behavior, communication, Embiidina, Embioptera, silk, subsocial, territoriality, temporal patterns, Theme software, webspinner

## Abstract

Substrate vibration communication is displayed by a variety of insects that rely on silk for shelter. Such signaling is often associated with territoriality and social interactions. The goal in this study was to explore the use of substrate vibration by subsocial insects of the little-studied order Embioptera (also known as Embiidina). *Antipaluria urichi* (Saussure) (Embioptera: Clothodidae) from Trinidad and Tobago, a large embiopteran, exhibits maternal care and facultatively colonial behavior. Previous observations suggested that they were aggressive while guarding eggs but gregarious when not. Egg-guarders in particular have been observed shaking and lunging their bodies, but to date these putative signals have not been recorded nor were their contexts known. Staged interactions were conducted in the laboratory using residents that had established silk domiciles enveloping piezo-electric film used to detect vibrations. Predictions from two competing hypotheses, the maternal territoriality hypothesis and the group cohesion hypothesis, were erected to explain the occurrence of signaling. Experiments pitted pre-reproductive and egg-guarding residents against female and male intruders, representing social partners that ranged from potentially threatening to innocuous or even helpful. Behavioral acts were identified and scored along with associated substrate vibrations, which were measured for associated body movements, duration, and frequency spectra. Signals, sorted by the distinct actions used to generate them, were lunge, shake, push up, and snapback. Egg-guarding females produced most signals in response to female intruders, a result that supported the maternal territoriality hypothesis. Female intruders generally responded to such signaling by moving away from egg-guarding residents. In contrast, pre-reproductive residents did not signal much, and intruders settled beside them. Theme software was used to analyze the behavioral event recordings to seek patterns over time and their association with signals. Long patterns of behavioral acts were associated with shakes, lunges, and push-ups, indicating that interactions were occurring between the residents and intruders as would be expected when communication occurs. The value of Theme software, as well as the relationship between signaling by *A. urichi* and the risks and benefits of coloniality, are discussed.

## Introduction

**Figure 1.**
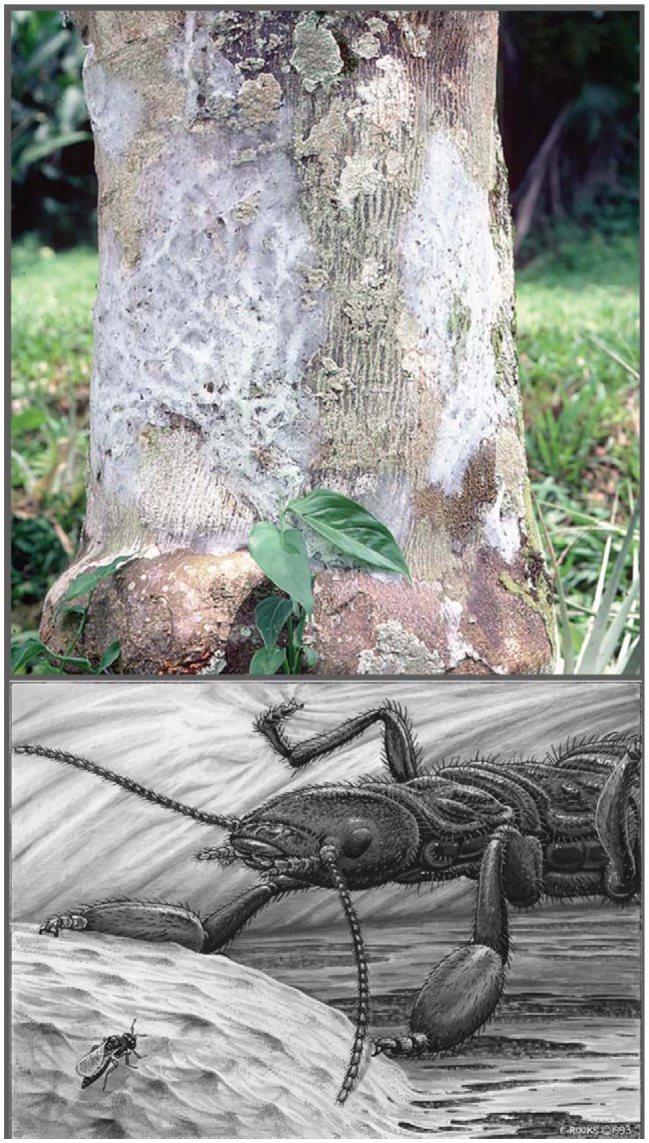
Silk of 2 colonies *of Antipaluria urichi* on lichen-festooned bark in a citrus orchard in Trinidad's Northern Range (top). The silk structure includes a sheet-like covering over tubular galleries that build up as individuals move from central resting positions to lichens where they graze at night. The adult (bottom) is shown within the silk guarding an egg mass (drawing by EC Rooks). In a typical pose, her middle legs are hooked into the roof-like silk covering. The bulge in the foreground is the egg mass (averaging 53 eggs), hidden by a covering of gathered materials topped with thick silk. A parasitoid wasp perched on the silk covering awaits a chance to dig in and probe for host eggs, which she cannot do in the presence of an egg-guarder. For scale, note that adult *A. urichi* females average about 1.6 cm in length. High quality figures are available online.

Animals living in groups benefit from certain enhancements, such as more effective defenses or the ability to build more formidable nests than is possible when solitary. They also can suffer costs, increased risks of disease and parasitism being high among those costs. For some species, the costs associated with group living rise and fall as parasites increase and decrease in abundance. The parasitism problem alone might preclude higher levels of sociality, as seems to be the case for *Antipaluria urichi* (Saussure) (Embioptera: Clothodidae; Figure 1), a subsocial insect whose populations are comprised of coexisting solitary and colonial females. For example, egg-guarding females are vulnerable to costs associated with close contact with conspecifics, but at the same time they can benefit from sharing silk with others. In response to shifts in consequences of group living, display behaviors may have evolved that allow individuals to express their willingness to cluster together. Preliminary observations suggested that the actions of shaking and lunging by *A. urichi* (Edgerly 1986) might function as displays expressing territoriality in a silk-sharing, colonial insect. Two goals for this study were first to quantify and contextualize the display behaviors of *A. urichi* to test an overarching research hypothesis that their displays act as communication signals, and second, to apply an analytical process developed to detect structure hidden within complex behavioral interactions. First a brief introduction to the order and a summary of observations that triggered the research questions and provided the rationale for the experimental protocols is presented.

### Who are the embiopterans?

Insects of the order Embioptera, a cosmopolitan group comprised of approximately 2000 species, are found mostly in warm regions (Ross 2000). A particularly outstanding trait is that females are neotenous; as such, they are wingless and soft-bodied, characteristics correlated with their secretive existence within tight spaces. In addition, they sport silk glands in their enlarged front tarsi and through a series of dance-like steps create silken domiciles and covered passageways on tree bark, under rocks, or within leaf litter (Video 1). *A. urichi*, for example, spins sheet-like silk over bark where they hide and forage on epiphytic algae and lichens (Figure 1). Their white silk ranges from small patches (less than 100 cm^2^) to extensive coverings (ex. 37,000 cm^2^ for one large colony) on trunks and major branches of large trees in rainforests of Trinidad and Tobago (Edgerly 1987a, b, 1988, 1994). Colonies consist of solitary females with their young or colonial females in various stages of reproduction. One survey of 44 colonies revealed that among the 57 adult females discovered, 36 were solitary while the remainder lived in groups of 2 or more (up to 9 egg-guarding females in one colony) (Edgerly 1987b).

**Video 1.**
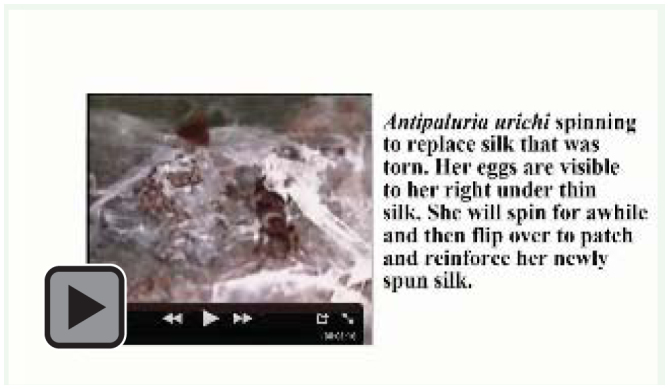
*Antipaluria urichi* spinning silk in the laboratory after her silk was torn. Her eggs are within the silk domicile and can be seen on her right. She spins by stepping with her front feet as multiple strands of silk issue forth from her tarsal silk glands. Click image to view video. Download Video

### Facultatively colonial: benefits and risks

For most of their lives, individuals of *A. urichi* benefit from the close proximity of others. Furthermore, they seek opportunities to share silk, as is evidenced by the common occurrence of “joiners” identified during a long-term field census (Edgerly 1987b) and in laboratory (Edgerly et al. 2006) and field experiments (Edgerly 1986). On the other hand, if females are guarding eggs (as depicted in Figure 1), over approximately 6 weeks, they potentially suffer by being near others similarly guarding eggs (Edgerly 1987b). This is because eggs of colonial females experience higher parasitism rates by scelionid wasps than their more isolated counterparts. When within a silk covering, parasitoids search for neighboring host eggs, thus putting colonial *A. urichi* at higher risk. Previous observations revealed that egg-guarding females were irritable in response to conspecifics that came close to them and they often vigorously shook their bodies (denoted as “lunge” in Edgerly 1987a) in a manner that resembled signals as seen in other insects, including *Clothoda* n. sp. (Clothodidae), a closely related embiopteran from Ecuador (Proaño et al. 2012). The lunges and shakes of *A. urichi* did not involve direct physical contact between the insects and did not appear to serve other functions such as travel or tending the eggs. Thus, the actions of egg-guarders appeared similar to territorial signals produced by the caterpillar *Caloptilia serotinella* when they defend silk shelters (Fletcher et al. 2006). *A. urichi* do not leave the immediate vicinity of their eggs, because if they do, the ubiquitous scelionid parasitoids will attempt to oviposit. Thus, egg-guarders might move away from their eggs to respond to intruding conspecifics, but quickly return to their eggs to repel scelionids (Edgerly 1987a). An egg-guarder's inability to leave her eggs opens up opportunities for conspecific intruders seeking residence because they can settle inside the silk covering but just out of her reach. Joiners add their own silk to the structure, thus expanding the protective covering. The potential advantage of living near one another, despite conflicts with egg-guarders, is that expansive silk provides a defense against predators (Edgerly 1994). Because siblings are known to disperse away from their natal silk as they approach adult-hood, a high degree of relatedness between colonial adult females is unlikely, although genetic relatedness measurements were not made in previous field studies (Edgerly 1987b). The impact of relatedness on social interactions also was not explored in the present investigation.

**Table 1.**
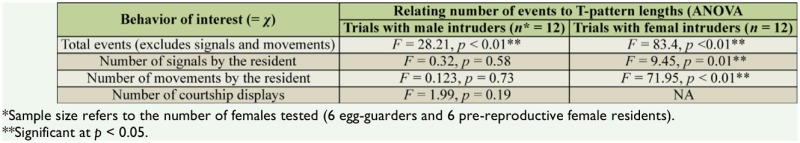
Results of regression analyses testing the relationship between number of behavior events (*x*) and the lengths of t-patterns (*y*) discovered by Theme software for interactions between intruders and resident *Antipaluria urichi.*

The resemblance between substrate vibration signaling in a wide range of insects (see Table 1 in Hill 2009; Table 1 in Virant-Doberlet and Cokl 2004) and that of *A. urichi* suggested that the lunging and shaking displays of *A. urichi* function as signals. The switch in tendency to lunge by female *A. urichi*, as documented in behavioral time budgets (Edgerly 1987a), also suggested that the incidence was dependent on a female's reproductive stage. Their expression of this shift provided an opportunity to test different hypotheses related to their increasing defensiveness during the time of their lives when negative consequences of living close together increase. Also potentially related to risk is the nature of an intruder. For example, a resident female might be more likely to signal if the intruder poses a threat to her eggs. Based on the authors' knowledge of *A. urichi* natural history, intruding males and females present very different threats. Females (mean length 1.6 cm; Edgerly 1987a) may be a greater threat to egg-guarders because they increase the risk of egg parasitism because a higher concentration of eggs might attract more scelionids. Males (mean length 1.18 cm; Edgerly 1987a) do not represent such a threat and in fact only live for a short period of time after reaching maturity because they do not feed during this time.

Silk-sharing *A. urichi* experience a decreased risk of predation as mentioned above (Edgerly 1994). Sharing silk by adults does not appear to be in response to the costs associated with the behavior of spinning, since these are low, but rather to the functioning of the silk, which is enhanced when many individuals contribute to its structural integrity and expansiveness (Edgerly et al. 2006). Furthermore, when females disperse they must walk out in the open. They experience a mortality rate of at least 25% due to predation (Edgerly 1986). This selective pressure might explain their tendency to enter pre-existing silk (Edgerly 1987b), and the safety provided by silk might counter-balance the risk of higher concentrations of egg parasites. In contrast to this embiopteran system, silk that is expensive to produce seems to be the reason why aggressive attacks are elicited when caterpillars attempt to join resident silk spinners in *Depressaria pastinacella* (Berenbaum et al. 1993).

### Display behavior and social interactions

A goal of this study was to conduct experiments to determine if vibratory displays act as signals, and whether displays and social inter-actions track the fluctuating benefits and costs of coloniality in *A. urichi.* Vibratory displays were identified and characterized, as has recently been done for *Clothoda* n. sp., another embiopteran (Proaño et al. 2012) and for many other insects that rely on substrate vibration for communication (for general reviews see Hill 2001, 2009; Virant-Doberlet and Cokl 2004; Cocroft and Rodriguez 2005). The scope of our study was expanded in this regard to document characteristics of male displays as a contrast to the female's, all of which involve shaking the body and/or moving the silk up and down.

Substrate vibrations function for group cohesion or for competition, hypotheses formulated and tested by Fletcher (2007), for example, in a study of communication in sawfly larvae. As for embiopterans, egg-guarding individuals observed as focal subjects in field colonies by Edgerly (1987a) lunged and shook more than adult females in other life stages (pre-reproductive or when with nymphs). Such evidence suggested that these vibratory displays served a territorial function, but experimental work was needed to systematically expose residents to various intruders. Two competing hypotheses with associated predictions were developed to guide the present experimental design. The naternal territoriality hypothesis (MTH) explains the incidence of vibratory displays as related to reproductive status of the actor, whereby egg-guarders engage in territorial behaviors that establish a safe zone around their eggs. Thus, the predictions following from the MTH were (1) adult females guarding their eggs will exhibit more vibratory displays than will pre-reproductives, (2) vibratory cues will serve to push intruders away from the vulnerable eggs, (3) adult female intruders will trigger more displays than males, which do not pose a threat, and (4) displays will occur when the intruder is near the eggs. However, if females are pro-social, as suggested by other observations in the field and lab, then vibratory cues might stimulate intruders to join. This argument led to a competing hypothesis, the group cohesion hypothesis (GCH), which states that vibratory behaviors act to alert individuals about the presence and/or position of others, and these cues elicit gregarious behaviors, such as settling nearby within shared silk. If female joiners can contribute silk to the structure in a helpful manner, pre-reproductive residents should also be sensitive to the sex of a joiner (males do not spin much whereas females do). If vibration displays serve either to push others away or to trigger cohesion, then female residents should be more responsive to intruders who represent potential social partners (GCH) or competitive threats to eggs (MTH). Males are largely irrelevant in both cases.

To characterize the vibrational displays of *A. urichi* and the behavioral interactions that triggered and followed them, temporal patterns of behaviors and positions of residents and intruders were evaluated. One prediction was that behavioral interactions of varying complexity would be triggered by promoting encounters between residents and intruders of different status. An ancillary prediction associated with the two hypotheses (GCH and MTH) was that agonistic interactions would be more complex when motivations of residents and intruders conflicted. Pre-reproductive residents might welcome female intruders and their interactions would be simpler because the intruder would quickly settle inside the silk. Alternatively, female intruders seeking to join the silk of an egg-guarder should trigger territorial reactions at each attempt made to settle nearby. A resident in this situation was predicted to produce vibratory cues embedded in complex patterns of behaviors as she engaged the intruder. In sum, a conflict of interest should be detectable as complexity in the interaction; the range of intruder-resident treatments were designed to promote this predicted distinction.

To discover how vibratory displays fit into a framework of associated behaviors and observable changes in the receiver *(sensu* Hebets and Papaj 2005), time event data were analyzed with Theme (Noldus Information Technology, www.noldus.com), which is software that detects temporal patterns in complex behavior (Magnusson 2000). Re-searchers in psychology (Haynal-Reymond et al. 2005), behavior (Arthur and Magnusson 2005; Kerepesi et al. 2005; Casarurubea et al. 2009, 2010), and neuroscience (e.g., Nicol et al. 2005; Horn et al. 2011), to name a few, have employed Theme to identify patterns in behavioral data. For example, Arthur and Magnusson (2005) used Theme to characterize the syntax of communication underlying interactions between male and female *Drosophila.* Readers are referred to these references to find more extensive explanations of Theme than provided here.

**Supplementary Figure 1.**
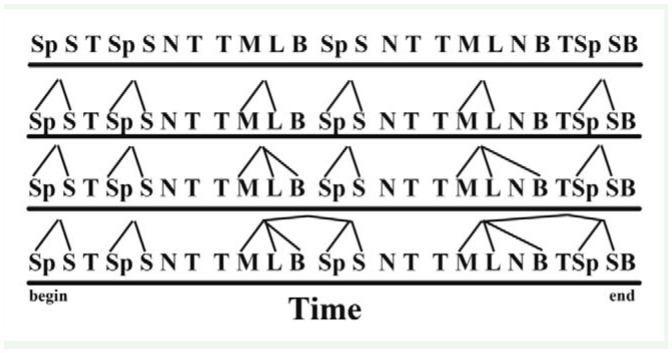
Illustration of systematic pattern detection by Theme software. The top line of symbols shows a string through time of hypothetical behavioral acts symbolized by letters, such as “Sp” for spin. The second line shows pairs of behavioral acts detected by Theme on its first pass as it searches for non-random patterns through time. Pairs of behavioral acts are treated as single units for a second pass, and so forth as Theme systematically detects longer and longer combinations. Illustration is based on Magnusson (2000). High quality figures are available online.

Patterns of behaviors over time, as detected by Theme (dubbed t-patterns by Magnusson (2000)), can be detected even when unrelated events occur in between components of the patterns. Theme uses a binomial test to determine statistically related patterns by first detecting associated pairs of behaviors. Those pairs then act as events as more complex patterns that contain them are sought, and this process is repeated, detecting longer and longer patterns until no more are found and the search process stops. A hypothetical example illustrating the hierarchical bottom-up detection process of Theme is provided in Supplementary Figure 1. Casarrubea et al. (2009) conducted an analysis of various methods for handling behavior data and found that Theme did not require *a priori* noise reduction or deletion of rare events. This is an advantage because display behaviors can be rare relative to the entire data set. The attributes of Theme were compelling because preliminary work suggested that patterns of behavior for interacting *A. urichi* were not easily detectable. Preliminary observations also suggested that when a male courts, females sit and “listen,” but when females “argue” over silk occupancy and territory, back and forth reactions result. Using Theme allows one to explore complexity, such as the prediction that displays of egg-guarders confronted by intruding females would appear within longer t-patterns than would courtship signals if the resident's signals are territorial and if a female intruder is contrary by attempting to settle within her silk.

Shaking and lunging exhibited by embiopterans closely resemble locomotory behaviors such as travel and U-turn because these actions all involve body displacement. However, the locomotory behaviors had such low amplitude that they were not detectable by the piezo-electric film used in our study, and therefore amplitude and frequency could not be compared. Although human observers can see the differences between the behaviors mentioned above, it might be true that any movement by a resident could be interpreted by an intruder as a cue of her presence. Theme provided a mechanism for statistically evaluating interactions between intruders and residents and how these might be associated with a resident's locomotory actions or to the substrate vibrations, which otherwise do not appear to serve any other purpose than as signals.

## Materials and Methods

### Cultures

Laboratory cultures were founded from hundreds of *A. urichi* collected as eggs, nymphs, and adults in the Northern Range Mountains of Trinidad in 1998. Colonies were drawn from 14 census sites along a south-north transect through the montane rainforest and subsequently mixed together in the lab. Additional field-caught webspinners were added in 2003, and in total the cultures contained hundreds of individuals, mostly immatures. They were maintained at room temperature (approximately 27° C) at Santa Clara University in 4 large plastic boxes (28 cm × 28 cm × 24 cm) filled with dry *Quercus agrifolia* Née (Fagales: Fagaceae) leaves, which form a matrix in which they spin silk. Romaine lettuce and lichens were added twice a week as food, and the boxes were misted with water. This method was adapted from Ross (2000), who has reared embiopterans in cultures since the 1940s.

### Establishing experimental residents

**Figure 2.**
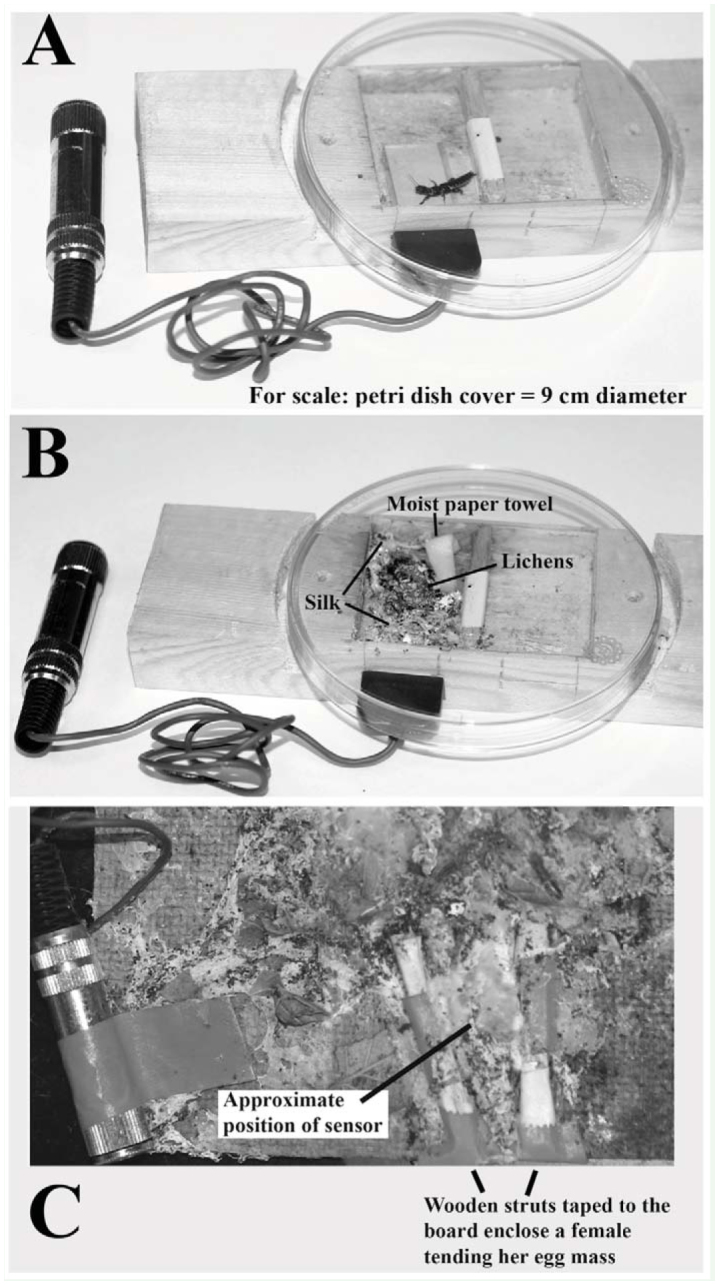
Experimental apparatus housing *Antipaluria urichi* females with piezo-electric film for detecting substrate vibrations. (A) Plywood block is carved to create a chamber sectioned off with taped struts in the middle to encourage the female to settle near the detector (slipped through a notch in the block). An adult is shown sitting in a new chamber. (B) Occupied apparatus showing silk, moistened paper towel, and lichen bits as food. To view interactions, a magnifier headset (5× power) and bright, angled lighting to illuminate the insects within the silk were used. Even with these aids, visibility was low. Disturbing the silk, however, was not the favored solution, and thus some measurements (like body displacement) were not possible for all signals. (C) Alternative set-up of masonite board where *A. urichi* were allowed to settle freely from their culture box. Egg-guarders (n = 4) were identified at the edges, and the piezo-film was slipped in next to their eggs. Wooden struts served to hoist the silk and to isolate the female and her eggs from other individuals, one of which is visible just above the right-hand strut. Plastic lids further isolated the residents. Black dots are fecal pellets, which the insects stitched into their silk. High quality figures are available online.

To elicit, capture, and characterize substrate vibrations, over a period of more than 3 years, a series of individual *A. urichi* adult females were placed in 1 of 3 wood chambers (2 cm wide × 4.5 cm long × 0.7 cm deep) carved into plywood blocks (Figure 2A, B). Moistened paper towel (to maintain humidity) and lettuce and sprigs of lichen as food prompted them to establish a silk domicile. The arena was covered with a plastic Petri dish lid, slipped into grooves in the wooden block, and hinged with tape so that it could be lifted during feeding. A female became an established resident when she spun thick silk above the floor of the chamber near a vibration sensor (piezo-electric film contact pickup (2.3 ×1.3 cm) SDT1 with audio jack by Measurement Specialties Incorporated (http://www.measspec.com)). Although these are standard methods (as an example see Fletcher et al. 2006), the process proved difficult because few females settled or spun silk near the piezo-electric film (perhaps because of the smooth plastic coating). The original plan was to test the same females before egg-laying (pre-reproductive) and during egg-guarding, but because of reluctance of females to oviposit, different individuals were tested for each treatment.

Overall, only 6 of 24 adult females recruited for the study established silk galleries in the plywood arenas, as pre-reproductive females and 2 others laid eggs in the plywood arena near the piezo-electric film. Because of low oviposition rates, the apparatus was modified in an attempt to encourage egg-laying. For this, lichens were stuck with egg-white glue to a masonite board (16 cm ×20 cm), and embiopterans in the stock culture were allowed to voluntarily move onto the board (Figure 2C). Four females ultimately affixed their eggs near the edge of the board, and piezo-electric films were slipped in next to their egg masses. Balsawood sticks served as struts alongside the eggs to create a frame for an arena where interactions were staged. A clear plastic lid was placed on top of the frame to enclose the resident, her silk, and her eggs. With the 6 pre-reproductive females and the 6 egg-guarders, almost 1,000 vibratory displays were elicited during the trials.

Sample sizes were similar to those presented by Fletcher et al. (2006), who characterized signaling in a caterpillar. Therefore, even though small, our sample of resident females appears adequate for research of this nature. Despite the difficulties of studying Embioptera, the differences between treatments were fairly strong, allowing detection of interesting patterns and behaviors and exploration of our research questions on the transient nature of territoriality and coloniality and how these behaviors related to signaling.

### Experimental trials

Once a resident established a silk domicile encompassing the piezo-electric film, she was challenged with intruders over a period of days. This technique of staging interactions is a method commonly employed to elicit signals (e.g., Yack et al. 2001; Fletcher 2007). Intruders were adult males or pre-reproductive females (non-gravid, recently eclosed adults and large nymphs). Female intruders were not in the same age cohort and thus were not siblings of the residents. They were selected randomly from 1 of 4 culture boxes without regard to relatedness. The final sample included 35 trials with egg-guarders and 18 with pre-reproductive adult females. The 6 pre-reproductive females were paired with only 1 male each. In contrast, individual egg-guarding female residents were tested repeatedly with male and female intruders because egg-guarders were likely to signal, providing substrate vibrations for recording and characterization, a major goal of this study. Supplementary Table 1 gives a full account of replicate numbers for each female resident. No individual intruder was tested more than once, but residents were measured repeatedly. Generally, no resident was tested more than once in a day, but a few were tested twice a day with a gap of at least 6 hr between trials.

During an interaction between intruder and resident, vibrations produced when the embiopterans shook or lunged were amplified through a K&K Sound Systems Pure PreAmp (frequency range of 10–30,000 Hz, www.kksound.com). Vibrations were converted to digital with a SoundTech Lightsnake Instrument (www.soundtech.com) to USB Cable (frequency response range: 20 Hz to 19.2 kHz) and recorded with Audacity Free-ware (version 1.2.6; audacity.sourceforge.net) onto a miniMac or iMac (www.apple.com). Audacity recorded at a sampling rate of 44,100 Hz. Amplitude and frequency spectra were analyzed with MATLAB (MathWorks, www.mathworks.com). Trials began when an intruder was introduced into the arena and ended after 30 min. Interactions were filmed using a Solid State Javelin camera with a TV zoom lens and recorded with a digital video recorder. The relative positions between the resident and intruder, their behaviors (see Supplementary Table 2 for detailed list), and co-occurrence of substrate vibrations were scored using an event recorder programmed with The Observer XT (version 10, Noldus Information Technology, www.noldus.com). Intruder positions relative to the resident were scored as next to, within a body length, or away if more than a body length apart. The intruder was scored as inside or outside the resident's silk. Substrate vibrations were named based on the actions displayed, and ultimately 5 types were identified. Displacement of the signaler's body during signaling was measured for lunge and snapback. To do so, the digital video was played with stop motion and the maximum forward and backward thrust of the signaler was marked onto clear acetate sheets held against the computer screen. The marks were measured with a ruler. Signalers had to be visible through the silk to be scored in this manner.

### Statistical methods

All statistical tests, including ANOVA, matched pairs *t-*test, and regression, were conducted with JMP Pro 10 by SAS Institute Inc. (www.sas.com). Mean values were used in statistical tests to represent the behavior of individual residents, who were typically tested with more than 1 intruder for each treatment.

Random numbers were used to select vibration events as the sample from a list including only those generated while an individual was directly on the sensor. Mean values for each female for lunge, push-up, shake, and snap-back were compared to mean values for courtship signaling for amplitude at peak frequency, peak frequency (Hz), and length (ms) using ANOVA.

For analysis with Theme, behavioral data files were exported from Observer XT into Theme 6. The analysis parameters were set as follows: for each replicate, patterns of behavioral events must have occurred a minimum of 3 times to be detected as a t-pattern and at a significance level *p* = 0.005 (a typical setting to achieve a strict threshold for identifying patterns). T-pattern length is defined as the total number of behavioral acts in the pattern. As a first step, the option within Theme was selected to shuffle the data 10 times and search for t-patterns. A matched pairs test in JMP was conducted by comparing the mean pattern lengths discovered in real data to the shuffled data for each female. If the differences are significant, then patterns are not due to chance. The number and lengths of patterns that occurred for each replicate were determined, and comparisons were made to test if egg-guarding females displayed more complex interactions than pre-reproductive females when confronted by an intruding female. The same comparison was made for trials with male intruders. The relationship between the number of locomotory actions, signals, and total events were also compared to pattern lengths to test the prediction that signals would be embedded in more complex interactions than would simple locomotory actions.

**Video 2.**
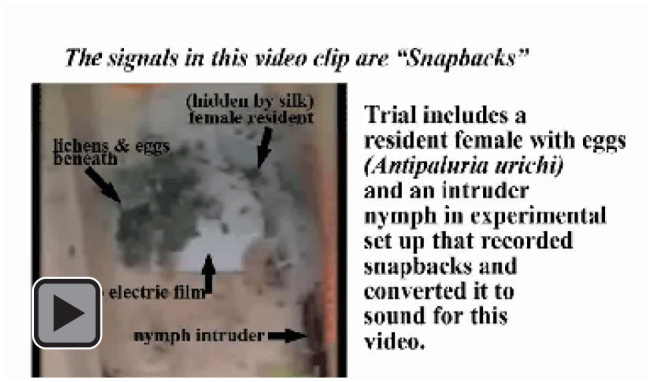
*Antipaluria urichi* resident egg-guarder in the experimental arena responding to a female intruder positioned just inside the silk tube at the lower right of the video. The resident is hidden by silk but her movements are obvious once she does a U-turn and faces the intruder. The resident displays a few snapbacks. Click image to view video. Download Video

**Figure 3.**
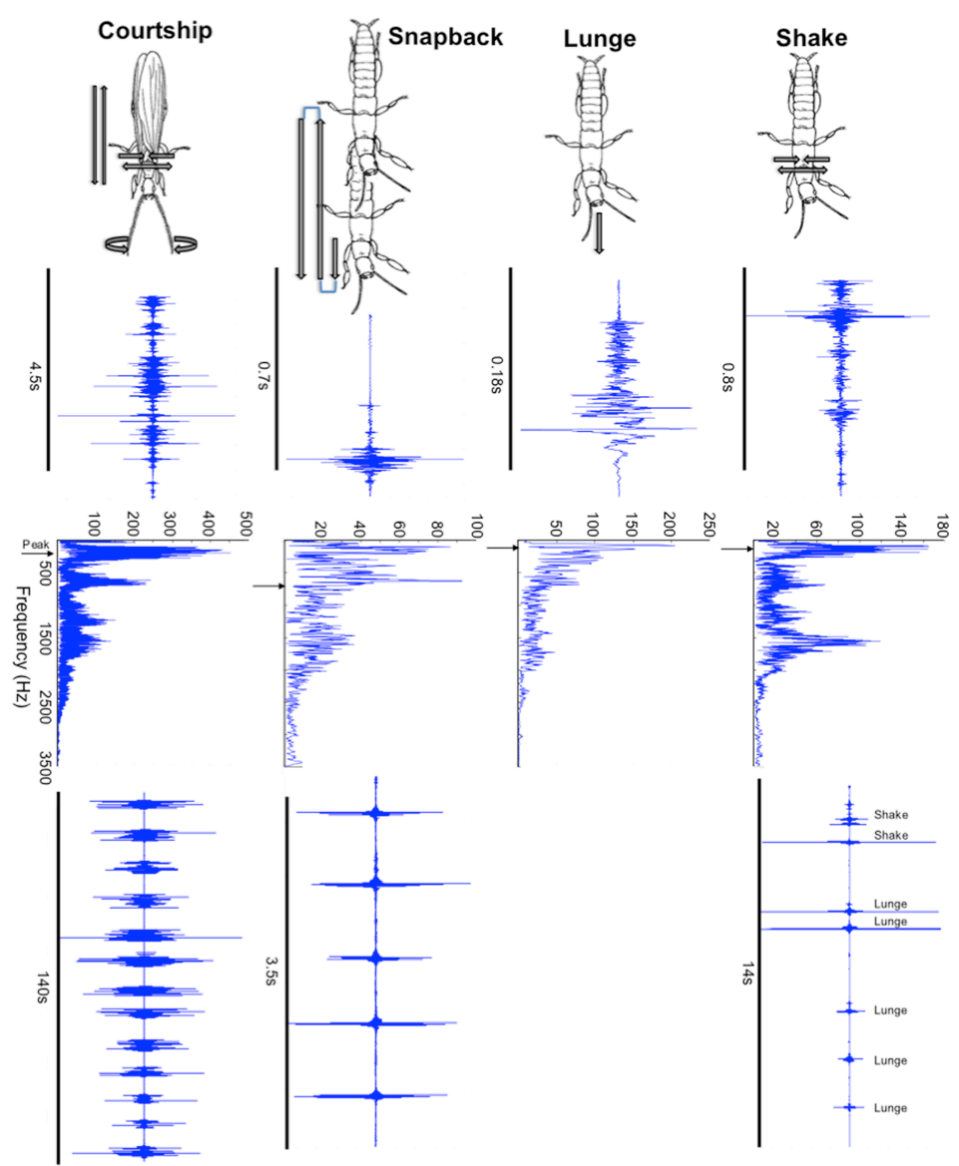
Four vibratory cues of female and male *Antipalura urichi.* Drawings show typical movements during each action. For each vibration type, from top to bottom, an oscillogram is shown of 1 burst, a frequency spectrum, and oscillogram of a series of vibrations (lunges and shakes are combined at lower right). Amplitude is relative for the oscillograms. Frequency spectrum for the courtship activity was determined for one pulse of a train of pulses, which typify courtship bouts as shown at lower left. Push-up is omitted because recordings were weak. High quality figures are available online.

**Figure 4.**
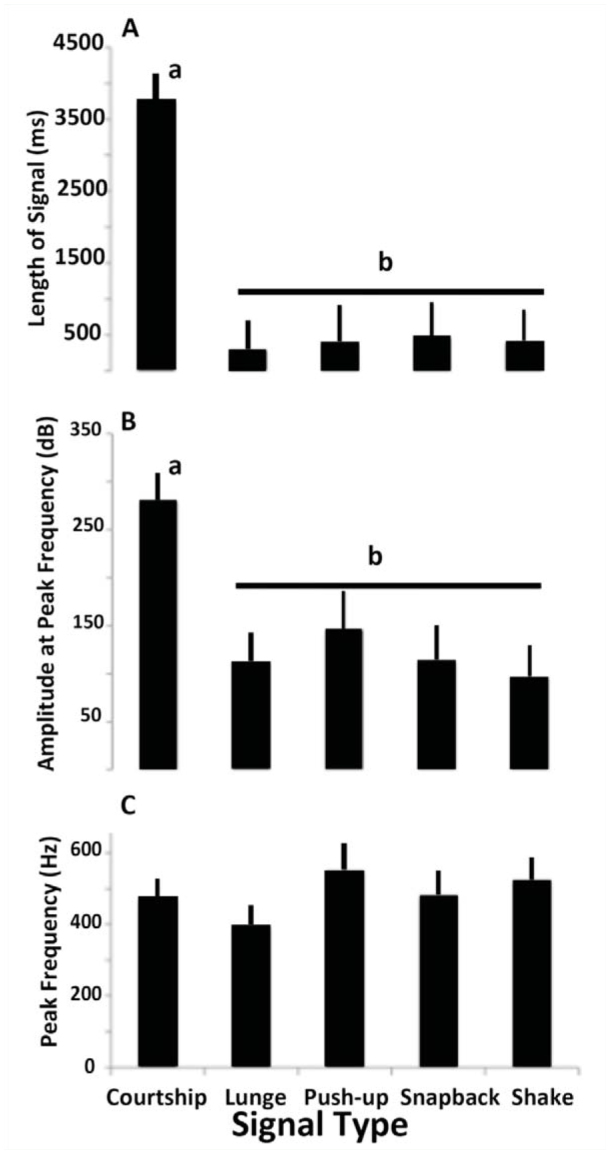
Characteristics (mean ± SE) of 5 signal types recorded during staged interactions between resident and intruders of *Antipaluria urichi.* Only signals displayed directly on the piezo-electric film were analyzed. (A) Length of the signal (ms). (B) Amplitude at peak frequency (dB). (C) Peak frequency in cycles per second (Hz). Sample sizes were courtship (n = 8), lunge (n = 7), push-up (n = 4), snapback (n = 5), and shake (n = 6). Different small letters indicate values that are significantly different, and similar letters show means that are not different, as indicated by Tukey's LSD following an ANOVA. High quality figures are available online.

## Results

### Characterization of substrate vibrations

Substrate vibrations displayed in 53 trials by *A. urichi* were identified and named based on distinct body movements. “Shake” is a rapid jittering of the body. “Push-up” is when the individual lifts the silk up and back down. “Lunge” is a forward push and slow return to the starting position. “Snapback” is a push forward followed by a rapid return to the starting position (see Video 2). Adult males did not exhibit any of these actions, but engaged in courtship signaling, a jumpy, rocking action involving the whole body, including twirling antennae, that was displayed in proximity to a number of the females.

Figure 3 illustrates the typical motions associated with the stronger signals, an oscillogram and spectrum of examples, and sample trains of signals. Shakes and lunges occasionally appeared in series, as shown. Snapbacks were quick and energetic and occurred in series. Courtship signals were produced in bouts, which some males repeated during the 30-minute trial. Figure 3 shows a 140 sec bout of courtship.

**Figure 5.**
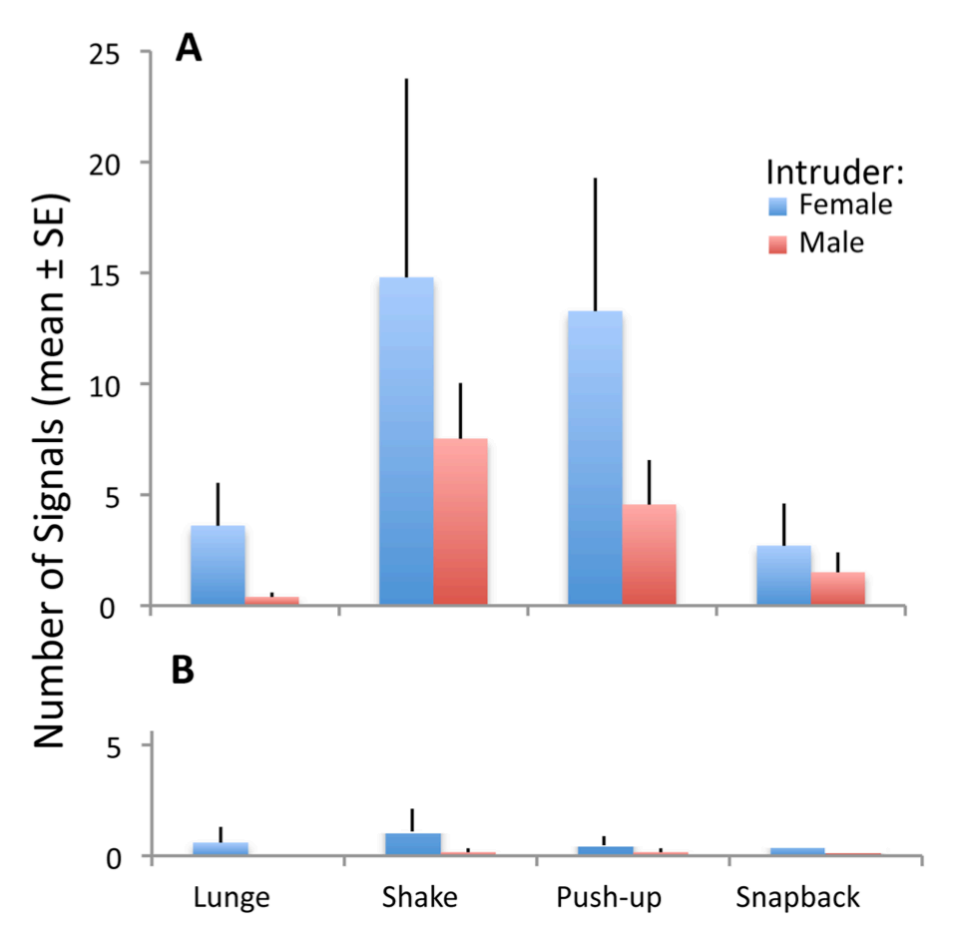
Number of signals of 4 types given by *Antipaluria urichi* residents that were either egg-guarders (A) or pre-reproductive females (B) in response to female or male intruders during staged interactions. Values shown are based on the grand mean of average scores for each female resident because they were tested repeatedly. See Supplemental Table 1 for details of each female and her signaling behavior in response to different intruders. One pre-reproductive female (named G in Supplemental Table 1 ) was excluded as an outlier from this analysis because her behavior was completely different from the other pre-reproductive females. She repeatedly signaled (especially with push-ups) female intruders, whereas all other pre-reproductives did not signal much if at all. High quality figures are available online.

Length of the signal (Figure 4A) and amplitude at peak frequency (Figure 4B) differed between male courtship and all female signals, but the female-generated vibrations did not vary as a function of these characteristics. Peak frequency (Figure 4C) did not differ significantly for the 5 signals. Even with courtship signaling removed from the ANOVA, body movements remained the only distinguishing characteristic of the 4 female substrate vibrations. Snapback and lunges are recognizable because of the forward displacement of the body (0.27 mm ±0.04 (SE) for lunge *(n =* 6) and 0.29 ±0.05 for snap-backs (*n* = 5)). Snapbacks also included a backward displacement, on average 0.28 ± 0.02 mm. The total displacement of the body was significantly different for these 2 lunging-type actions (*t* = 3.96, *p* < 0.01). Because of small replicate sizes and the variability and complexity of interactions, whether intruders responded differently to the 4 female- generated signals was not tested.

**Figure 6.**
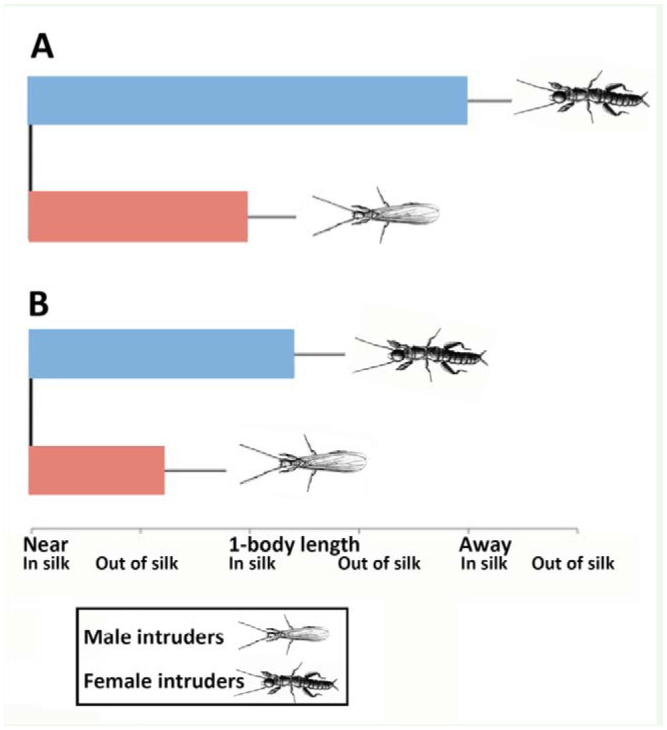
Average positions (± SE) at the end of 30-minute trials where female resident *Antipaluria urichi* were exposed to male or female intruders. Residents were either egg-guarders (A) or pre-reproductive adult females (B). Positions were based on a 6 point scale with “Near, In silk” being awarded a score of I and ranging to 6 for “Away, Out of silk”. Grand means for each resident-intruder combination, shown here, were determined by averaging the means for each female resident because most of them were challenged multiple times by female and male intruders. High quality figures are available online.

### Substrate vibrations and behavioral activity in context

**Supplementary Figure 2.**
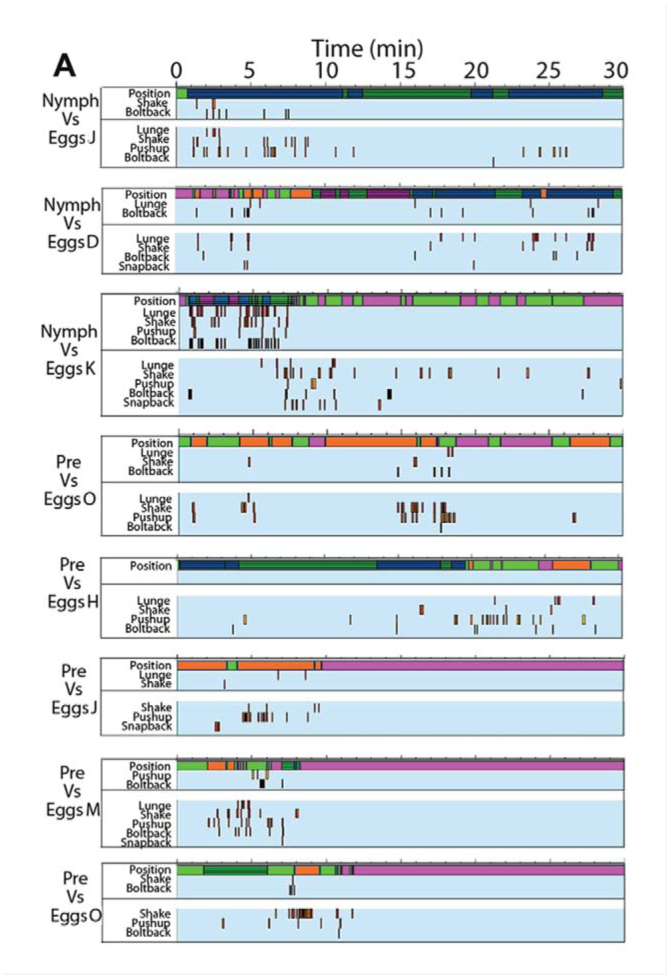
Time-event plots of relevant behaviors for trials with *Antipaluria urichi* where intruders and residents interacted. Samples from each resident-intruder combination are shown to illustrate variability in the responses observed. Color bar at top of each panel shows relative positions of intruder to resident: hot pink represents times when the intruder was away from the resident but inside the silk (with stripes) or completely away and outside the silk (solid pink). See legend in section B. (A) Egg-guarders paired with female intruders. (B) Egg-guarders paired with males. (C) Pre-reproductive residents paired with females (above) and with males (below). High quality figures are available online.

Intruder responses greatly influenced incidence of substrate vibrations, but interactions between residents and intruders were unpredictable and varied (as revealed in samples of time event graphs in Supplementary Figure 2A, B, C). An intruder was free to abruptly respond (bolt back) or not (sit still) to a resident, or to leave the silk or stay. In some cases, the resident signaled when the intruder was near the resident. Intruders also signaled, but never as often as residents. In other trials, intruders ran off after a flurry of signals from the resident. In others, an intruder persisted in coming near the resident who displayed vibratory cues in response. At times, the intruder would leave and return, eliciting a snapback, shake, or lunge from the resident. Nine of 12 males exhibited courtship behaviors. Egg-guarders typically displayed vibrations in response to males, however the rate varied greatly. For example, 1 female signaled once and the male followed this with courtship. In comparison, another female signaled many times (with shakes, lunges, and push-ups) while the male was outside the silk, but once near her he began courting and she stopped. The rate that males courted varied from high to very low. In contrast to egg-guarding females, pre-reproductive resident females did not tend to signal in response to intruding males. Trials where intruders were female showed more of a range. For example, one pre-reproductive female was reactive to intruders, whereas the other residents produced few vibrations or none at all, even though intruders often sat next to them. Eggs hatched for 2 females previously scored when egg-guarding; they were challenged with intruders. Before hatch, both females produced multiple signals in response to adult female intruders in 3 replicates each (32 ±14.27 (SE) and 19 ± 12.25). After hatch, these rates fell (1 ±0 and 6 ±6.3 respectively over 3 replicates for each female). Because of small sample sizes, statistical tests were not applied, but these results suggest that changes in reproductive status is an important factor in tendency to signal and as such are worthy of future studies.

An ANOVA, with main effects of resident status and intruder type (male or female), revealed that overall, egg-guarding females produced significantly more lunges (*F* = 6.9, *p* = 0.02), shakes (*F* = 5.25, *p* = 0.03), snap-backs (*F* = 4.1, *p* = 0.05), and push-ups (*F* = 8.77, *p* < 0.01) than did pre-reproductive females, with push-ups significantly different only when an outlier is removed from the set (see details of pre-reproductive female G in Supplementary Table 1). One trial of an egg-guarder was also removed from this analysis because the male intruder did not enter her silk and she repeatedly signaled him until he did enter approximately 20 m into the trial. If all signals are summed, the difference between egg-guarders and pre-reproductive females is highly significant (*F* = 11.23, *p* < 0.01). Female intruders elicited significantly more lunges (*F* = 7.1, *p* = 0.02) and push-ups (*F* = 4.9, *p* = 0.04) from residents than did males (Figure 5A, B). Snapbacks (*F* = 0.005, *p* = 0.94) and shakes (*F* = 1.74, *p* = 0.2) did not differ for trials with male and female intruders (Figure 5A, B). For this and all other statistical tests, nymphal and adult female intruders did not differ and were combined in the analysis. In addition, interactions were not significant and were removed from the ANOVA.

The position of the intruder significantly affected the likelihood of signaling by the resident. This analysis focused on egg-guarding females because few pre- reproductive females signaled. A matched pairs *t-*test revealed that significantly more signaling occurred for each resident when either a male or female intruder was within (mean number of signals = 34.9 ±12 (SE) and 13.3. ±4.2 respectively), rather than more than, one-body length (1.53 ±0.77 and 0.06 ± 0.06) of the resident (one-tailed *t* = 2.88, *p* = 0.02 for comparing rate of signaling in the 2 positions in response to female intruders and *t* = 3.18, *p* = 0.01 for male intruders).

Resident status and intruder-type had a significant effect on the final positions of the actors such that intruders associated with egg-guarders ended up further away from her than when they interacted with pre-reproductive residents (Figure 6). A matched pairs *t-*test showed that individual egg-guarders ended their trials with males closer to them than female intruders (one-tailed *t* = 4.71, *p*< 0.01), but this relationship did not hold for trials with pre-reproductive residents where intruders did not differ significantly in their behavior as a function of gender (one-tailed *t* = 1.22, *p* = 0.15).

Although signals were more commonly displayed by egg-guarders, resident status alone does not relate to overall behavioral activity displayed within a trial. The mean number of movements displayed by the resident was not significantly different between egg-guarders (24.1 ±5.15) and pre-reproductive females (14.07 ±6.2; *t* = 1.25, *p* = 0.24). All behavioral events excluding movements and substrate vibrations also were not different (148.6 ± 28.6 for egg-guarders = 115.6 ±21.8 for pre-reproductive females = 0.92, *p* = 0.38).

### Analysis of substrate vibration and behavioral interactions using Theme software

**Figure 7.**
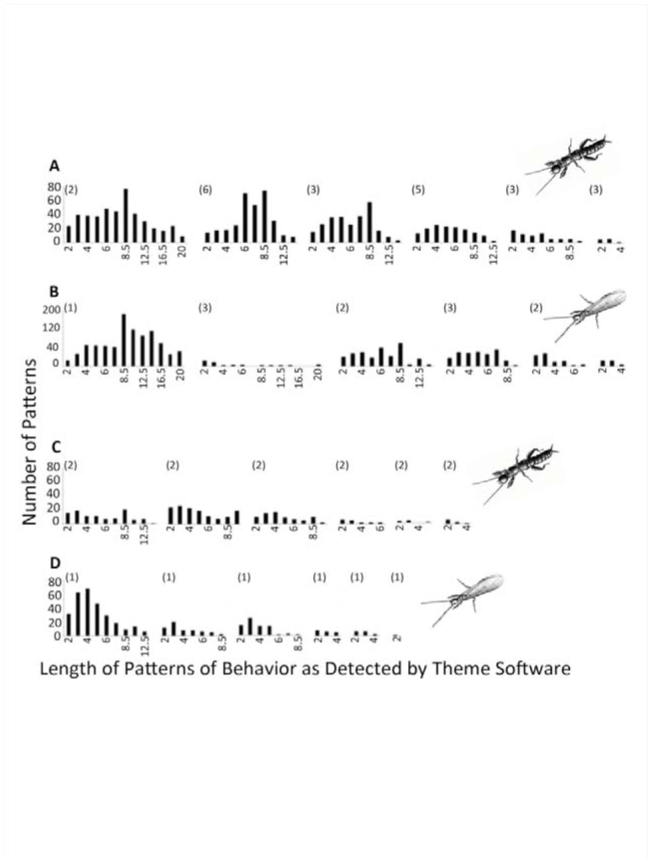
Results of analysis with Theme software showing the number of t-patterns of each length detected within trials for each resident female *Antipaluria urichi,* either egg-guarders (A and B) or pre-reproductive females (C and D). The intruders in staged interactions were either females (A and C) or males (B and D). Pattern length is based on the number of behavioral elements detected in the pattern. Numbers in parentheses (above and left) in each histogram show the number of replicates for each female. The histograms are aligned with the trials with the longest t-patterns to the left and shortest to the right. Where there is more then 1 trial per female, mean values are shown. Details of all trials are shown in Supplementary Table 1. High quality figures are available online.

The patterns of behavior detected by Theme in the real data sets (Figure 7) were significantly longer for all resident females when they interacted with intruding females (mean = 3.95 ± 0.61 (SE)) and males (3.6 ±0.43) than those in shuffled (randomized) data sets (female intruders = 2.07 ±0.23; for males = 1.77 ±0.33; mean difference between real and random for female-intruder trials = 1.525, paired *t*-test = 5.28, *p* < 0.01, *n=* 12; for male trials = 2.18; paired *t-*test = 4.06, *p* < 0.01, *n* = 12). Mean pattern lengths in trials with male intruders were significantly related to total events over-all but not to number of signals or movements made by the resident or incidences of courtship signaling by the male (Table 1). In contrast, pattern lengths were related to the number of signals given by the resident if the intruder was a female. Total events without signaling or movements and movements alone also were significantly related to pattern length in these trials.

**Figure 8.**
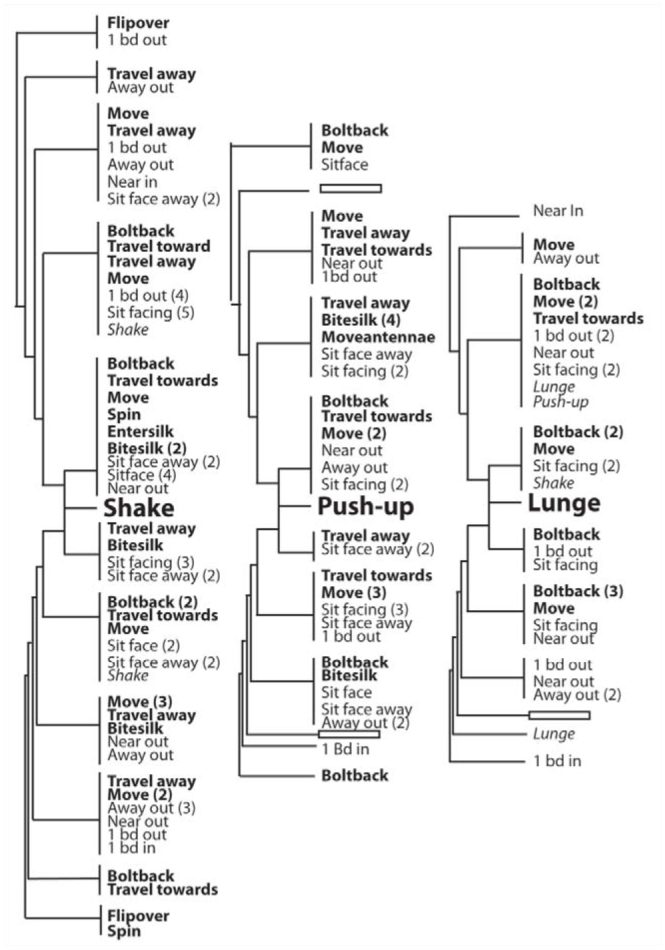
Connections between regularly occurring behaviors and signals given by resident egg-guarding females (n = 6) of *Antipaluria urichi* as detected by Theme software. Resident behaviors were excluded to highlight intruder responses to signals. The illustration shows a tree graph similar to that depicted in Supplementary Figure 3. Behavioral acts displayed above the signal names preceded the signal and those below followed. Boldfaced words are behavioral acts, relative positions are shown with plain text, and italics show signals given by intruders. Numbers in parentheses next to these actions show how many times they showed up in the network connection output tables of Theme. A first-level connection is a tighter connection between behaviors and the signals, shown as the first branch coming from the signal name in the center of the illustration. More diffuse connections are further away but still detectable in patterns of behavior. High quality figures are available online.

Signals were typically elicited by shifts in position of the intruder, as can be seen in the list of behaviors surrounding shake, lunge, or push-up as the center-points of t-patterns containing those actions (Figure 8). An analysis called Network Connections by Level in Theme uncovers regular connections between events as the number of steps between behavioral acts of each female intruder and the different substrate vibrations within patterns. Behaviors linked by lower level connections are those that occur next to each other in a t-pattern; these are shown as the first branches surrounding the name of the substrate vibration in the illustration. Courtship and snapback are not shown because very few behaviors connected to these actions. As can be seen in this descriptive analysis, behavioral acts that preceded or followed signals were highly variable, making it a challenging task to discover regular patterns of specific actions and reactions.

High variability between trials for individual female residents can be traced to variation in the amount of time spent interacting for some residents and intruders. For example, some intruders settled away from the resident for much of the 30 min, while others interacted repeatedly. To evaluate the question as to whether the structure of interactions differed in response to the resident's body movements or to substrate vibrations, an analysis was conducted to focus on the trial for each female when she produced the most signals, which were those trials where the intruder repeatedly interacted with her. Seven of 12 resident females (those that signaled) were evaluated in this test. The rationale for this analysis follows Arthur and Magnusson (2005), who used pattern length as a test for syntax of behavioral acts for communicating male and female *Drosophila.* For replicates where each resident signaled at her highest rate, mean pattern length was significantly and positively related to the number of signals for the subset of patterns that included signals (R^2^ = 0.64; *F_1,6_* = 8.97, *p* = 0.03) but not for the subset that in- cluded the residents' locomotory movements (*F_1,6_* = 0.002, *p* = 0.96). If resident movements were analyzed similarly, length of patterns was not significantly related to the number of movements for the subset of patterns with signals embedded within them (*F_1,6_* = 0.99, *p* = 0.36) or the subset with just movements (*F_1,6_* = 0.14, *p* = 0.73).

## Discussion

In their review of complex signal function, Hebets and Papaj (2005) noted that the more we know about communication, the more we see that signaling involves complex behavioral routines and often incorporates more than one signal. Probing interactions in *A. urichi* revealed a vibration communication system that in many ways matched this description. Staged interactions elicited multiple types of substrate vibrations, and analysis with the software Theme revealed that patterns of behaviors associated with signals, although not easily characterized, were lengthy and intricate. Responses depended on both the context and the motivations of the participants. Despite the complexity of the interactions, general features of their signaling emerged, especially as it related to reproductive status and sexual behavior. In general, the MTH gained support from the analytical approach. Signals can be sorted to territorial alerts and challenges (push-up, shakes, snapbacks, lunges), which were typically produced by egg-guarding females, to courtship by males. Below, we will evaluate the data in light of our predictions, and compare this system to that of other insects that use substrate vibrations in communication.

### Who signaled, and what were the consequences?

Overall, the differential rate of signaling supported the hypothesis that egg-guarders are territorial, an attribute expressed as an increased rate of signaling as predicted by the MTH. Only 1 of 6 pre-reproductive females signaled often. Comparing responses of egg-guarders to the different intruders (male or female) allowed us to determine if egg-guarders were generally irritable and territorial. In fact, their responses aligned with another prediction of the MTH stating that female intruders, but not males, posed a risk, and the resident's response to them was “stay away.” In turn, a female intruder's response was often to remain in the silk, albeit at least a body length away from the egg-guarding resident. This result gave credence to the supposition that intruders were motivated to adopt ready-made silk, as has been noted in field censuses discussed in the introduction. An absence of substrate vibration, as seen in most of the trials with pre-reproductive females, was associated with the intruder settling next to the resident and indicated that shaking, lunging and snapbacks, in particular, were not functioning in promoting group cohesion as expected if the GCH explained the existence of these signals. In sum, the sub-strate vibrations mapped more to territoriality than to cohesion. Questions still remain. For example, intruding gravid females (not tested herein) would potentially be less inclined to share silk with resident egg-guarders even if they could, because their imminent egg-laying might put them at risk of parasitism if they settle next to the eggs of another.

Males responded differently to female residents and vice versa, as expected if males were seeking mates rather than a silk dwelling to share. Egg-guarders often signaled males when they bit into the silk and entered, but they stopped signaling when the male courted or sat near by. These findings align with the expectation that males were not a threat and that signaling relates more to territorial behavior.

When challenged, signaling of 2 post-hatch mothers dropped precipitously compared to when they were guarding eggs. This result matches field observations that mothers with nymphs did not produce substrate vibrations, although none of those females were experimentally challenged with intruders as in this laboratory study. Field mothers with nymphs also produced significantly more silk once their eggs hatched (Edgerly 1987a, 1988) and as such might benefit from sharing silk spinning. More experimentally staged interactions are needed to further test the idea that post-hatch mothers share silk spinning and benefit from coloniality in this regard. In sum, the shifts detected in the tendency to signal in response to intruders in staged encounters suggested that signals align with territoriality as females transitioned from gregarious (pre-reproductives with few signals) to territorial (egg guarders with multiple signals) and back to gregarious (post hatch with few signals). Ultimately, egg parasitoids may act as selective agents favoring females that spread out (Edgerly 1987b), while predators select for females that share silk (Edgerly 1994). As proposed at the outset, status-specific signaling might provide a mechanism for dealing with the conflicting challenges associated with social behavior and life stage in *A. urichi.*

Spats over occupancy are common for insects that rely on silken dwellings. Other silk spinning insects are not tolerant of joiners, and residents signal when intruders attempt to enter silk shelters (for examples, see Yack et al. 2001; Fletcher et al. 2006). When Scott, Matheson, and Yack (2010) staged encounters between hook-tip moth caterpillars, they saw clear-cut winners and losers. These researchers noted that silk is valuable, and it is adaptive for wanderers to inspect silk structures for occupancy. For *A. urichi,* wanderers in search of habitation will enter silk structures despite eliciting territorial signals of egg-guarders. Mostly, they settled inside the silk but outside the zone around the eggs. As for the caterpillars noted above, *A. urichi* intruders did not push out residents, although it is interesting to note that some of them displayed shakes and lunges.

### Characterizing signals *of A. urichi*

*A. urichi* signals were in the range for peak frequencies of substrate vibrations recorded for other insects (see Supplementary Figure 4) and were closest to *Umbonia crassicornis* (Cocroft 1999) and to those of another embiopteran, *Clothoda* n. sp. (Proaño et al. 2012), in the same family as *Antipaluria,* which is not surprising given that the body movements that generated the signals closely resembled those of *Clothoda.*

Many insects signal by transmitting vibrations through plant structures, such as stems and leaves (reviewed in Cocroft and Rodriguez 2005; Hill 2009). *A. urichi,* in contrast, moves silk, which forms a roof above her. The stance of the embiopteran may allow them to detect displacement of the silk because they hook the tarsal claws of their middle legs up and into the silk as shown in the drawing (Figure 1). Because this work was limited to less expensive equipment that was too bulky for incorporating into silk, only movements made by individuals standing on a sensor were record- ed. Other workers studying silk dwellers, such as spiders (Masters and Markl 1981), rely on laser-doppler technology because displacement can be detected, even if subtle, without disturbing the silk. Future work on embiopterans warrants these methods, especially now that a variety of signals have been detected (for other examples of laser-doppler recordings of signals see Scott et al. 2010 and Ericksson et al. 2011).

The information encoded in signals might show an intruder that the resident is physically fit and able to fight if challenged. Yet, although the signals varied behaviorally, piezo-electric film did not detect differences for peak frequency or amplitude. Even with this lack of differences, snapbacks were particularly striking because they involved sharp and energetic forward and back motions, often displayed in series (Video 2). Surprisingly, push-ups, which seemed less forceful and possibly derived from actions associated with silk spinning, did not differ from snapbacks in amplitude. When an embiopteran travels in her domicile, she often pushes up against the silk to stretch it. She also often pushes the silk up when she is spinning. So, why claim that the push-up functions as a signal? Push-ups move the silk up and down, and the magnitude of the displacement is not subtle to the human eye (although this was not able to be measured because of the angle of the camera). During staged interactions, egg-guarding females displayed push-ups, but pre-reproductives rarely displayed this action. It seemed that when an intruder approached an egg-guarder, a push-up alerted her to the resident's presence. The complexity of the interactions, as detected by Theme, left unanswered questions about the possible different functions of the signal-types such as push-ups and snapbacks. Comparison with the embiopteran *Clothoda* n. sp. is instructive because the actions displayed during signaling were very similar in appearance, so much so that they were given similar names: lift silk, snapback, and shake. *Clothoda* produced signals with different spectral characteristics and also modulated their signals in response to different intruders, and these were detected with the same equipment (Proaño et al. 2012) used herein except for the arena construction. Why the present study on *A. urichi* did not emulate these findings leaves questions about the sensitivity of the equipment when used in arenas composed of wood, as well as about the mechanisms of sensory detection of the signals.

### What Theme revealed about communication in *A. urichi*

Because signals are obvious body movements, other movements, such as U-turn and travel, are perceived differently, even though these actions also move the silk near the resident and intruder, because knowing the number of travel-type movements did not allow prediction of length of patterns of behavior, whereas number of signals was related in a positive way. This result gives support to the hypothesis that the vibrational cues identified by us are communication signals.

Analysis with Theme revealed that despite displaying long t-patterns, behavioral responses of intruders to female signals were not predictable. Instead, signaling by an egg-guarder was generally associated with a high level of reactivity on the part of a female intruder but not necessarily with specific behaviors. Comparing males was instructive because analysis with Theme revealed that neither courtship signals nor movement were related to pattern length, lending support to the idea that egg-guarders were interacting with female intruders in a more complex manner. T-patterns containing female signals often contained behaviors related to movement by the female intruders. Furthermore, incidences of movement and signaling were positively related to pattern length. When intruders moved, they triggered signals, and signals triggered them to move. Intruders also would change direction relative to the resident. These results aligned with the operational definition of a signal, including the criterion of “an observable change in behavior of the receiver” (Hebets and Papaj 2005), but the observable change for the embiopterans was fairly diffuse.

If a female were displaying territorial behaviors near her egg mass, intruders should move in response. Some patterns showed the intruders entering silk or sitting facing the resident immediately following a signal. They may have sought to join the other individual who became apparent because of its signaling. Shakes and push-ups were the most common signals and were associated with the most complex “conversations” between the resident and the intruders. Theme exposed some meanings of the signals, but many questions remain. Sometimes female intruders left the silk, even quite rapidly, but mostly they stayed a body-length or more away from the resident. Linking t-patterns to the end results of the interactions showed that an egg-guarder producing lunges, shakes, and push-ups, might be saying, “I am here and I want you to move away from my eggs.” The exact function of individual signals remains a question for future work, and we find we are in the same position as past researchers trying to interpret the honey bee's ambiguous “shaking” signal. Ultimately, in that case, Nieh (1998) employed a series of clever experiments to determine its function, and found that the meaning of shaking depended in part on the receiver's point of view. Applying lessons from Nieh's study might help in planning future experiments to fine-tune the search to understand the subtleties in embiopteran signals.

**Continued:**
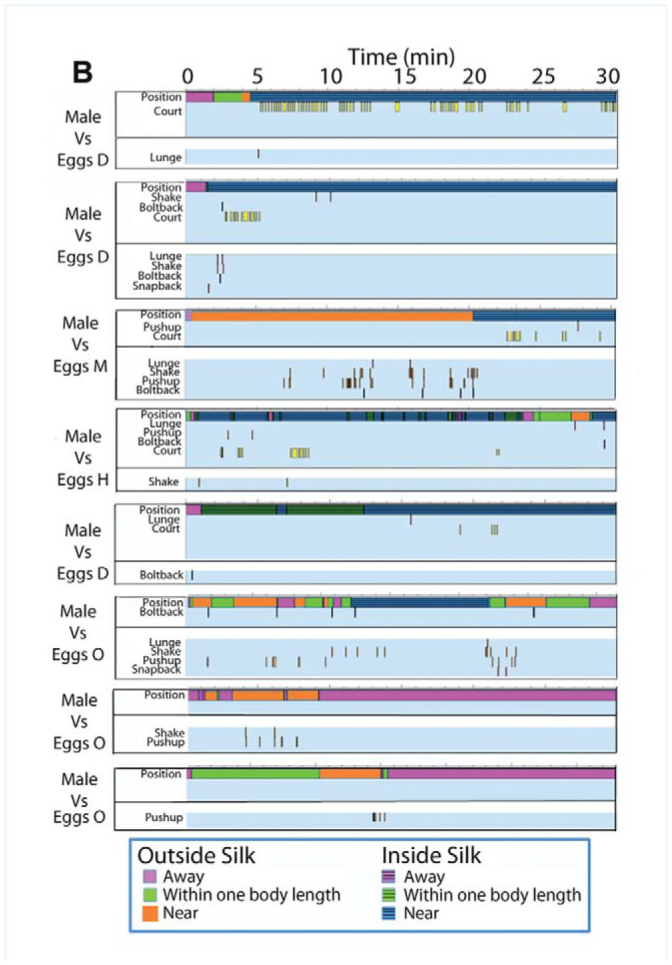

**Continued:**
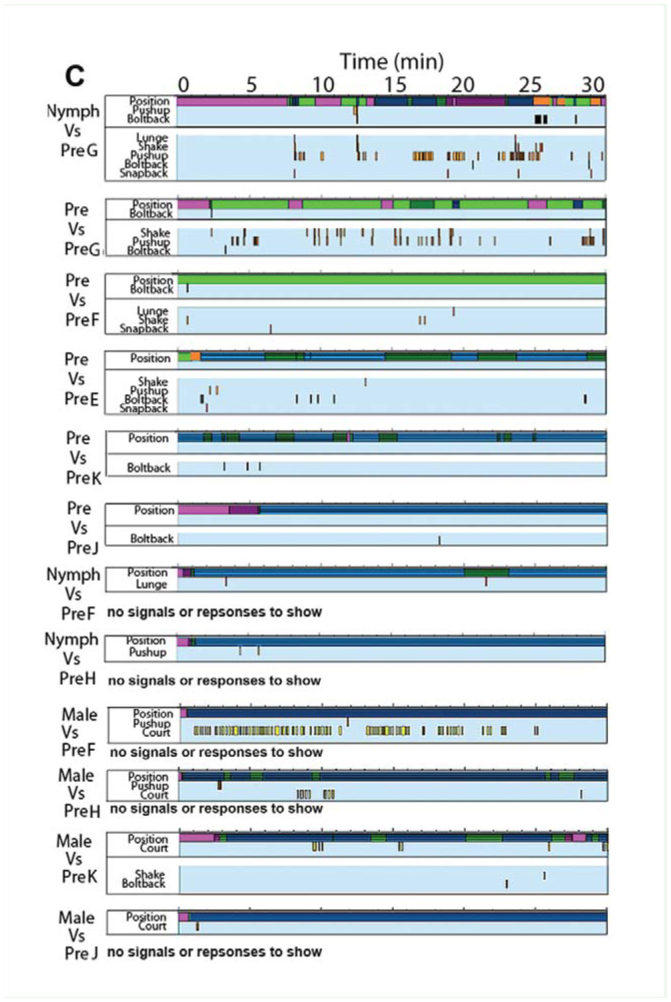

**Supplementary Figure 3.**
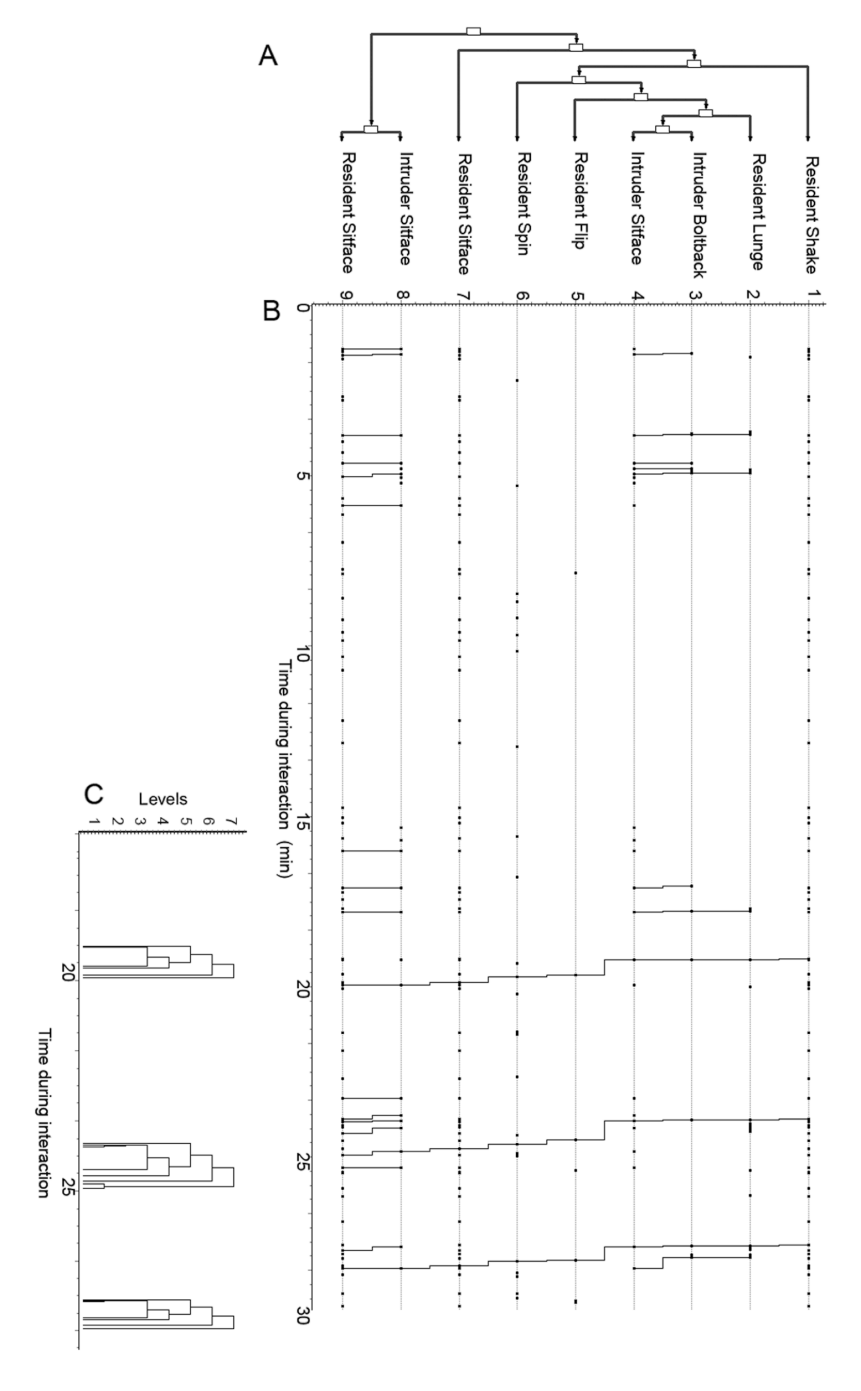
T-pattern from a 30-min interaction between a resident egg-guarder and a nymph of *Antipaluria urichi.* The t-patterns shown are produced by Theme software as tree graphs (A), connection graphs (B), and occurrences on the time-line (C). This example shows a resident “lunge” and intruder “boltback” followed by “sitface” (this 3-act t-pattern was detected 7 times). Parts of a longer t-pattern repeat, with the longest series including 9 behavioral acts repeating 3 times in its entirety. The illustration demonstrates the different ways of displaying non-random t-patterns detected by Theme. Theme counts the branches of the tree shown in A as “levels” between the behavioral act of interest and the associated behaviors. During this trial, a t-pattern with 9 behavioral acts occurred 3 times, plus numerous occurrences of snippets of this t-pattern throughout the trial. High quality figures are available online.

**Supplementary Figure 4.**
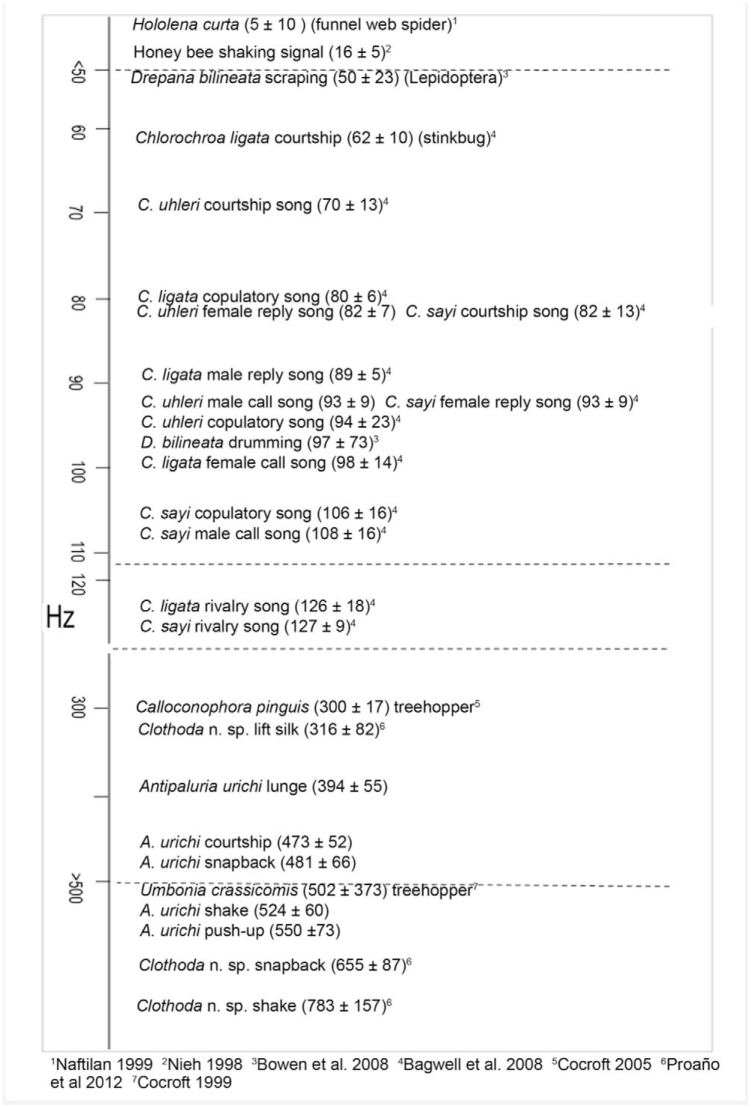
Frequency (Hz; mean ± SD) of a sample of published signals produced by insects and 1 spider with signals of *Antipaluria urichi* shown for comparison. All are substrate vibration signals except for the honey bee's shaking signal wherein worker bees shake another bee. Sample sizes for A*. urichi* signals are given in the caption for Figure 4. High quality figures are available online.

**Supplementary Table 1.**
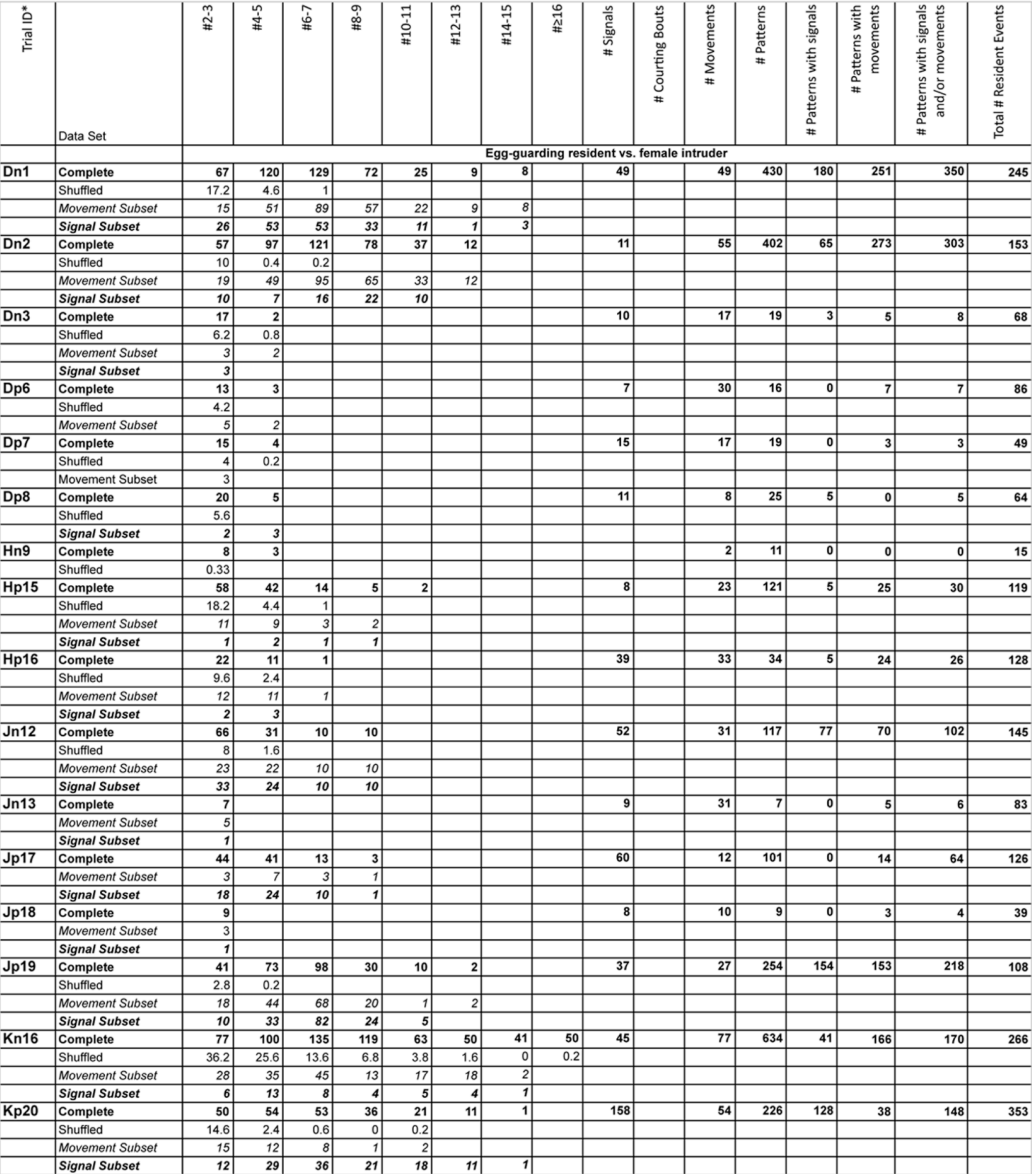
Number of t-patterns detected by Theme software in real (bold font) and randomized (plain font) data, number of signals generated by female *Antipaluria urichi* for each replicate, and length of patterns with movements (italic font) and signals (bold and italic font). The trial ID shows the residents ID, the intruder-type, and the replicate number for that intruder-resident combination. For example, Dn3 refers to Female D, a nymphal female intruder, and the third replicate with this combination.

**Continued:**
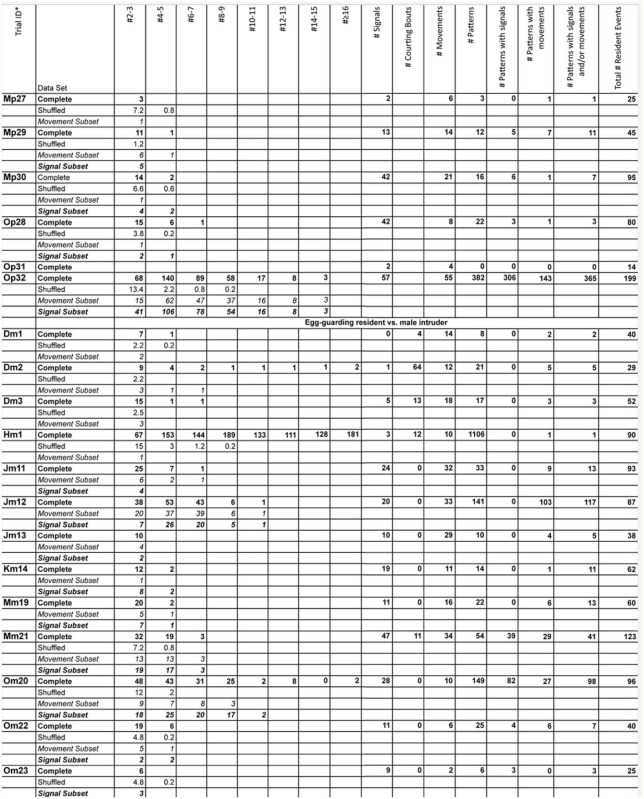

**Continued:**
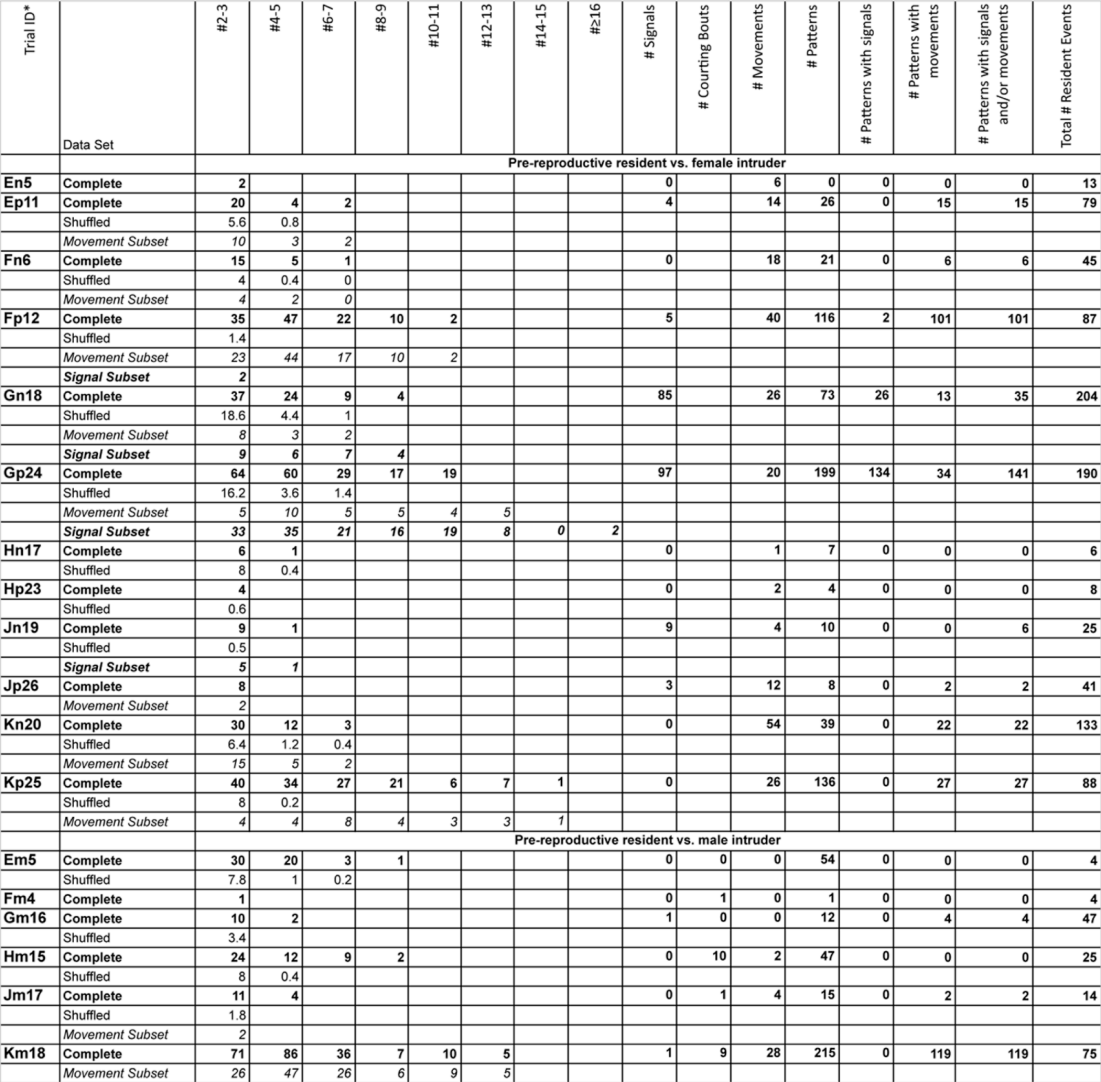

**Supplementary Table 2.**
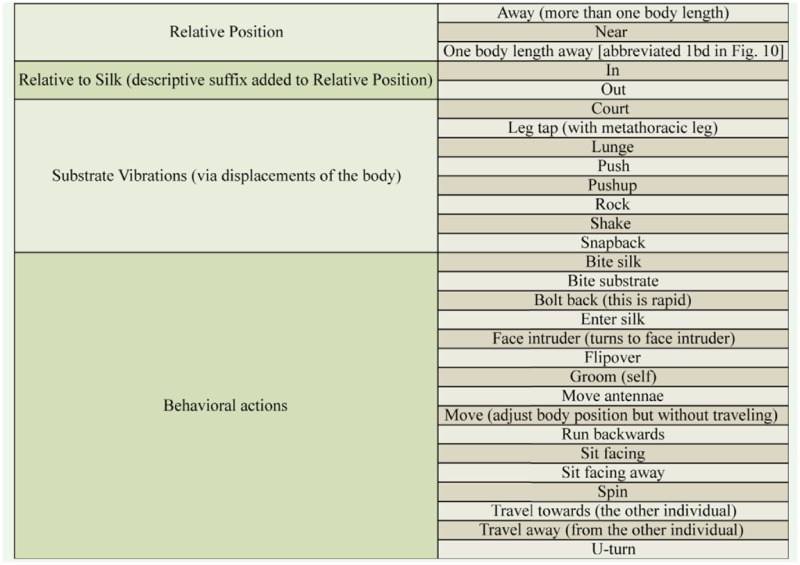
Elements of behaviors and positions scored with event recorder programmed with Observer software (description if needed in parentheses).

## Abbreviations

**GCH**,: group cohesion hypothesis
**MTH**,: maternal territoriality hypothesis
